# Simulations of silver-doped germanium-selenide glasses and their response to radiation

**DOI:** 10.1186/1556-276X-9-594

**Published:** 2014-10-29

**Authors:** Kiran Prasai, David A Drabold

**Affiliations:** 1 Department of Physics and Astronomy, Condensed Matter and Surface Science Program, Ohio University, Athens, OH 45701, USA

**Keywords:** Radiation damage, Chalcogenide glass, Dosimeter

## Abstract

Chalcogenide glasses doped with silver have many applications including their use as a novel radiation sensor. In this paper, we undertake the first atomistic simulation of radiation damage and healing in silver-doped Germanium-selenide glass. We jointly employ empirical potentials and *ab initio* methods to create and characterize new structural models and to show that they are in accord with many experimental observations. Next, we simulate a thermal spike and track the evolution of the radiation damage and its eventual healing by application of a simulated annealing process. The silver network is strongly affected by the rearrangements, and its connectivity (and thus contribution to the electrical conductivity) change rapidly in time. The electronic structure of the material after annealing is essentially identical to that of the initial structure.

## Background

Chalcogenide materials are among the most flexible and useful in current technology. Certain GeSbTe alloys are the basis of phase change memory technology [1] (now a credible alternative to conventional FLASH memory) and DVDs [2]. Amorphous Se is the active element for digital x-ray radiography [3], and metal-doped chalcogenide glasses are among the best known solid electrolytes or ‘fast ion conductors’ [4] and form the basis for another quite promising class of FLASH memory devices, ‘conducting bridge’ memory. The basic science of the material is just as interesting as the other phenomena such as the optomechanical effect [5] and photomelting [6]. Recently, a new application has emerged: the use of chalcogenide glasses for the detection or sensing of radiation (a dosimeter) [7,8]. The electrical conductivity is found to be well-correlated to radiation dose [9]. With annealing, the damage is readily reversed so that the device may be reused. This important discovery is presently empirically understood, suggesting the need for theoretical research both to understand the basic process and to aid in optimizing the materials for future device application.

In this paper, we undertake the first simulation to understand the atomistics of the response of chalcogenide glasses to highly energetic events. Like many other challenging material problems, we find it helpful to use multiple methods, in this case both empirical potentials, and *ab initio* techniques. We also have taken advantage of the contributions of others, such as the use of an appropriate ‘heat bath’ to handle the excess thermal energy after the thermal spike [10]. We detail the disordering process from a knock-on event to the subsequent recovery process. We show that the spike is indeed reversible upon annealing and discuss the electron states near the Fermi level - those responsible for the changes in (electronic) conduction after radiation exposure. The picture that emerges is that the electronic transport is significantly determined by the connectivity of the Ag subnetwork. Thus, if clusters percolate through the entire system, we have a network of nanowires that provide a low resistivity. As these nanowires break, form, or otherwise change, the carrier transport changes accordingly.

This paper is organized as follows. First, we discuss the formation of a suitable model of the material. Next, we track the damage wrought by a thermal spike and its healing. The electronic properties are discussed briefly and conclusions are provided at the end of the paper.

## Methods

### Model formation

Simulations have been widely used to characterize amorphous materials and satisfactory *ab initio* models of GeSeAg glasses for various compositions have already been reported [11,12]. Molecular dynamics (MD) simulation is a natural approach to simulate high-energy processes because MD offers detailed trajectories of the atoms as the system evolves after the radiation induced event. The pitfall of MD is that it is only as good as the force field used and it is as computationally costly as it is detailed. Furthermore, large models are needed to realistically simulate radiation events in any material and this greatly increases the computational cost.

Many simulations of radiation damage have been presented, with varying details and system sizes ranging from 446 to 2.3 million atoms; the following is a highly incomplete list [10,13-15]. Early simulations applied many approximations like the binary collision approximation (BCA) [16], linear interactions [16,17], and others to reduce the computational demand. Clever algorithms and parallel machines have enabled full simulations on large models [15]. We used the potential of Iyetomi et al. [18] to model the interatomic attractions. This potential is simple in its form containing a Coulomb interaction term, charge-dipole interaction term, and a short range repulsion term, and yet commendable in its ability to predict wide range of properties of this material.

The primary model of a silver rich glass (with stoichiometry close to that used for a detector) was fabricated as follows. We used a cubic supercell containing 5,184 atoms with periodic boundary condition to represent bulk Ge _3_*Se*_9_*Ag*_4_ (the cell had 984, 2,888, and 1,312 atoms of Ge, Se, and Ag, respectively). The model described in this work is obtained by using the melt-quenching method [19]. Starting from a randomly placed collection of atoms, we performed 10 ^5^ steps of MD with constant NVE. Then the atoms were given random velocities corresponding to a macroscopic temperature of 5,000 K and were allowed to equilibrate for another 10 ^5^ steps. The system was then cooled to 1,200 K over 3.8 × 10 ^5^ steps and equilibrated at 1,200 K for another 10 ^5^ steps. The system was then cooled to 300 K over 0.75 × 10 ^5^ steps and equilibrated at 300 K for 10 ^5^ steps. Finally, the system was relaxed using a conjugate gradient algorithm. The MD simulations described in this work were performed using the classical molecular dynamics simulation package LAMMPS [20]. A time step of 1 fs was used throughout, except when variable time steps were required.

**Figure 1:**
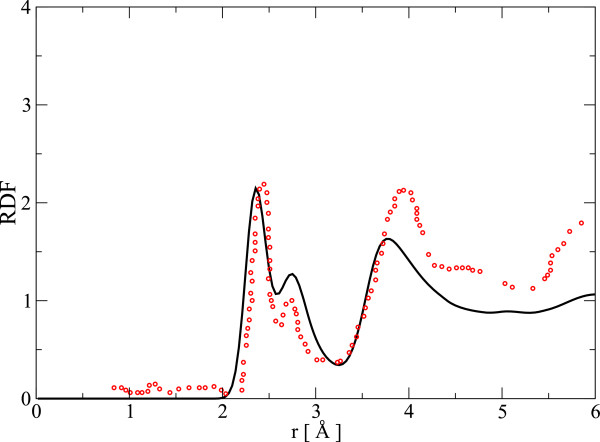
**RDF: models and experiment.** The total RDF of our model compared with experimental values for same composition (the red circles) from reference [21].

**Figure 2:**
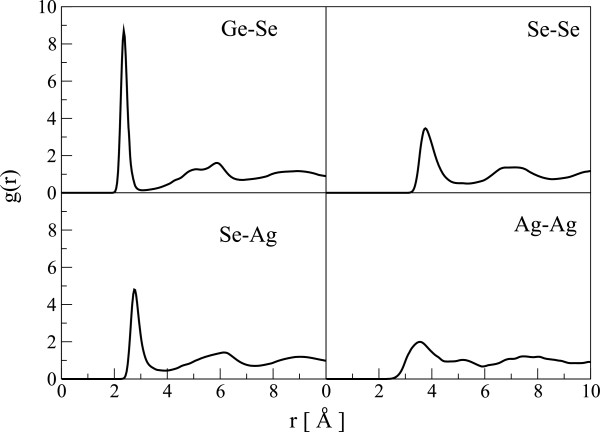
**Partial correlations.** The partial pair distribution function, *g(r)*, of our model. Note the weakly defined correlation of Ag-Ag interaction.

This model faithfully reproduces many features of the material (see also reference [18]). The total radial distribution function (RDF) given by this model is in reasonable agreement with the experimental RDF [21] including a two-peak first neighbor feature in *g*(*r*) (see Figure 1). Our model also reproduces the experimental nearest neighbor distances of Ge-Se, Se-Se, and Se-Ag correlations [21]. It overestimates the Ag-Ag correlation distance, but this can be understood considering the broad peak of Ag-Ag pair correlation function (see Figure 2).

**Figure 3:**
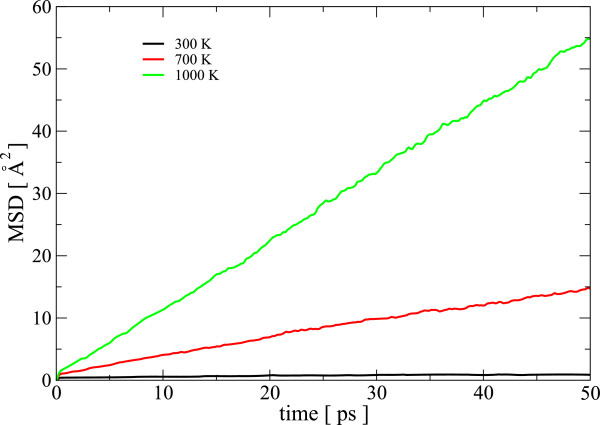
**Silver dynamics and diffusion.** Mean squared displacement of silver at different temperatures. The size of the simulation box is 50.86 Å.

The first peak of the partial RDF of Se-Ag displays a companion alongside the first peak in total RDF. The Se-Ag interatomic potential has a shallow minimum near the Se-Ag correlation distance, whereas all other interactions of Ag-atoms are repulsive. As a result, Ag atoms are very mobile in the network and Se atoms see high coordination with respect to Ag. The exceptional mobility of Ag atoms with respect to the host atoms is a widely reported phenomenon [11,18,22], and the basis of many applications, and even the accelerated crystallization of phase-change memory materials [23]. The mean-squared displacement (MSD) of atoms calculated for our model also predicts the high mobility of silver at all temperatures below the melting point of the material. Figure 3 shows the MSD for Ag atoms at different temperatures. The diffusion coefficients and conductivity calculated using the total MSD of the system compare favorably with the corresponding experimental and *ab initio* values reported for most similar system as ours (see Table 1).

**Table 1:** Diffusion coefficients and ionic conductivity

	**D(cm**^***2***^**/s)**			**Conductivity (Siemens/cm)**		
	**This work**	**Ref **[11]		**This work**	**Ref **[11]	**Expt **[24]
300 K	4.05e-7	1.16e-8		0.0989	5.3e-4	7.5e-5
700 K	2.33e-6	1.20e-5		0.2436	0.235	0.0657
1,000 K	6.84e-6	2.53e-5		0.501	0.347	0.2584

### Damage simulation using thermal spike

We carry out a thermal spike simulation using the model above. The damage inflicted on a material by high-energy radiation starts with a sudden transfer of kinetic energy from the incoming particle to an atom or a group of atoms that happen to suffer a collision with the incoming particle. For incident particles of energy in the range of MeV, the first interaction with the atoms on the target is entirely ballistic and the detailed role of interatomic potential between the impinging projectile and the target can be neglected. So, following Rubia et al. [14], we modeled the onset of radiation damage by igniting a thermal spike at the center of the supercell. We defined a sphere of radius 2.5 Å located at the center of the supercell, i.e., at d/2, d/2, and d/2, where *d* is the size of the cubic supercell to receive the thermal spike. For the particular configuration we modeled, this sphere contained two atoms. These two atoms were given an initial velocity consistent with 1 MeV of energy, and the rest of the atoms were assigned a velocity distribution associated with a temperature of 300 K. These conditions are thus intended to mimic a damage event at 300 K with the center two atoms representing the primary knock-on atoms (PKAs). The system was then allowed to evolve. For an isotropic material like GeSeAg and energy of PKA being as high as MeV, the direction of the initial velocity of the PKA should have no observable effect on the damage production.

**Figure 4:**
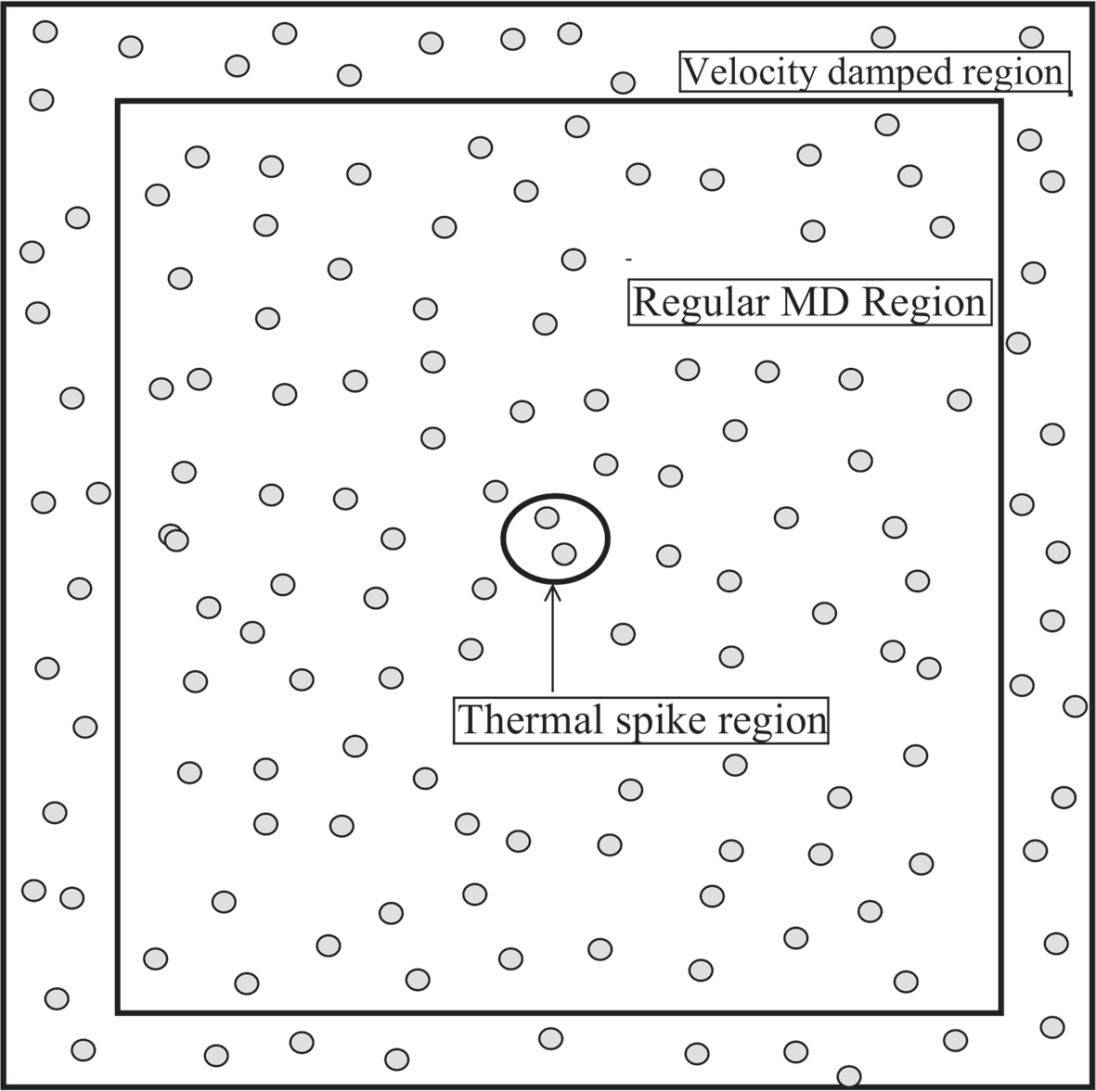
**Schematics of simulation box.** Diagram showing the regions of a simulation box where the thermal spike was modeled (the central circle), where the velocity rescaling was applied (the outer boundary), and where the normal molecular dynamics was performed.

In view of the large velocity imparted to the atoms and the unknown behavior of the empirical potential under extreme conditions, we used a variable time step as in [25]. To avoid the diverging cascade of damage from bouncing back from the boundary, we used a damped outer layer of 0.5 Å thickness. The velocities of the atoms falling in this boundary region were rescaled at every dynamical step [26]. The schematic diagram of the simulation setup is shown in Figure 4.

## Results and discussion

**Figure 5:**
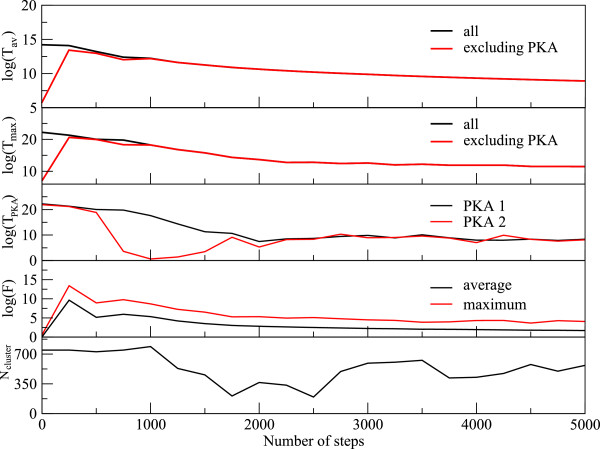
**Evolution of the thermal spike.** The logarithm of the average temperature (top box), logarithm of temperature of the hottest atom in the system (second box from top), logarithm of temperature of PKAs (third box from top), logarithm of force on the atoms (fourth box from top), and the size of largest cluster in the system (bottom).

**Figure 6:**
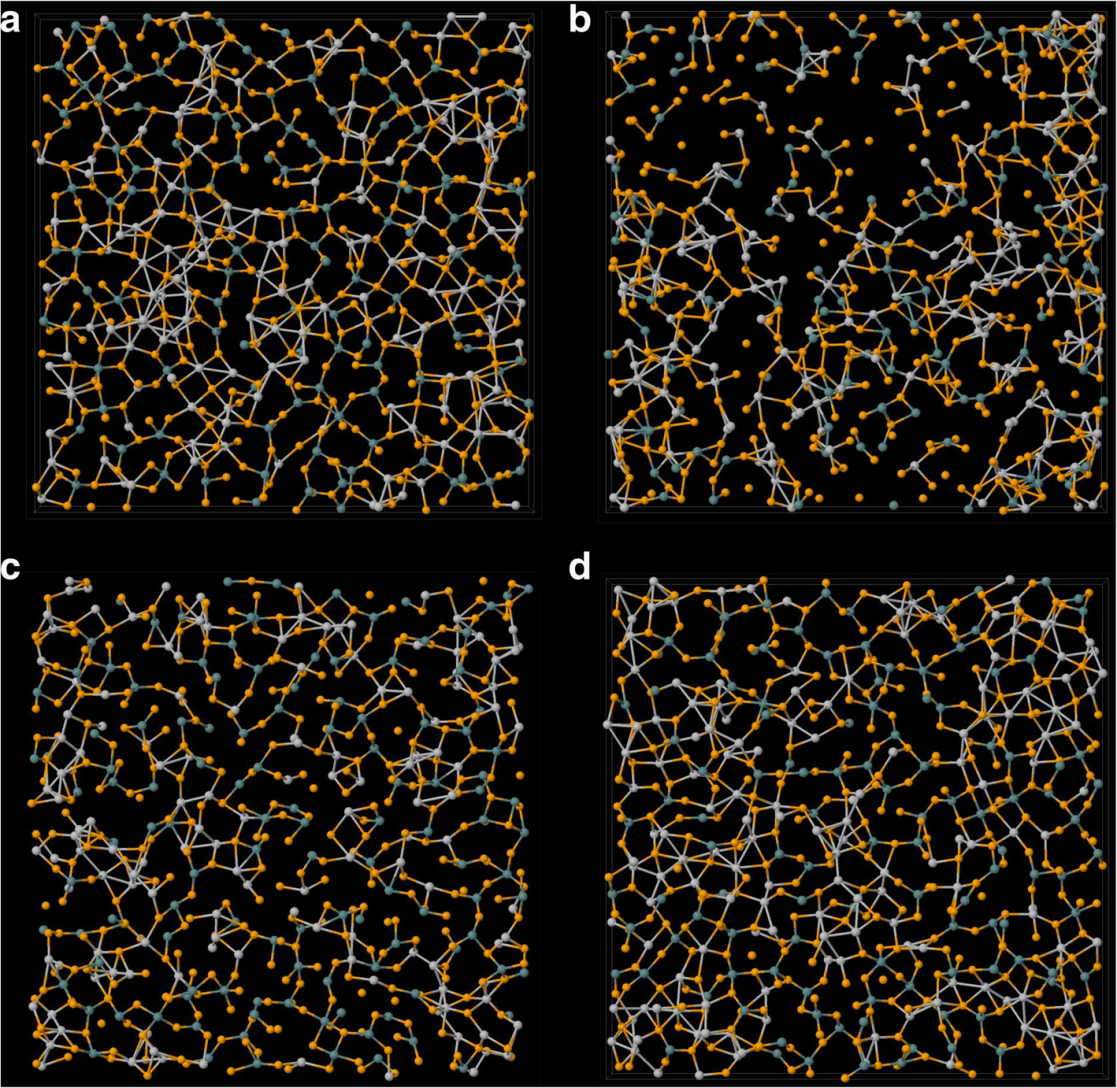
**Damaged snapshots.** Snapshots of central 6 Åof the simulation box **(a)** before the event, **(b)** at 2.25 ps after the event, **(c)** at 10 ps after the even, and **(d)** at 50 ps after the event (fully equilibrated). The temperature drops below the melting point at around 12 ps. Color nomenclature: Ge=blue, Se=orange, Ag=white.

**Figure 7:**
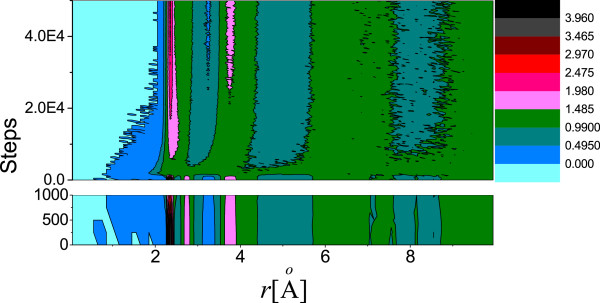
**Evolution of RDF.** The temporal change in total RDF of the system after the damage event. The features at the beginning are largely recovered after approximately 20,000 steps in the detonation-healing process. The RDF values for the first 1,000 steps after the detonation are highlighted in a separate band at the bottom. Note the peak values in the beginning and loss of the second peak of the first coordination.

**Figure 8:**
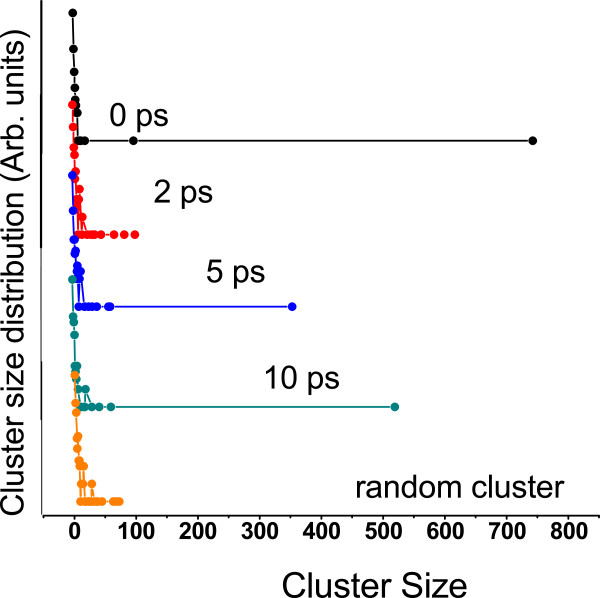
**Cluster size distribution.** The distribution of different sizes of Ag-clusters in system at the beginning, at 2 ps, at 5 ps, at 10 ps and in a random collection of Ag atoms. Vertical axis is in shifted logarithmic scale.

Immediately after the detonation of a thermal spike at the center, the hot atoms’ trajectories resemble a projectile, and they pass through the network transferring huge amount of momentum to the atoms along their trajectory. These recipients of momentum get knocked from the network and, in turn, start moving like a projectile, thus creating a cascade. At this stage, the velocity of the PKA is highly non-canonical as indicated by the observation that temperature of the system excluding the PKA lags behind the temperature of the whole system for a brief period of time (see Figure 5). An animation tracking the positions of atoms and the average temperature of the system reveals rapid local melting. The average temperature of the system remains above the melting point of GeSe_3_Ag (approximately 1150 K, [21]) for 11.9 ps. To visualize radiation damage inflicted on the network, we present snapshots of a slice of the supercell containing central 6 Å of the simulation box at different times after the detonation (Figure 6). The damage is most conspicuous at 2.25 ps, and the image clearly shows the formation of voids and internal surfaces. At 10 ps, the system can be seen returning to its initial structure as also indicated by first peak of total RDF gaining height around that time.With the onset of local melting, the system loses its short-range order. The temporal evolution of the RDF in Figure 7 shows an interesting recovery. The short-range order can be seen evolving continuously with the first peak gaining height and the first minimum continuously deepening. The evolution of second peak follows. The hump in the first peak originating from Se-Ag coordination, however, does not reappear as late as 50 ps.To investigate the effect of radiation events on the phase separation of Ag atoms, we performed a cluster analysis of Ag atoms over the entire evolution of damage. We defined a cluster as a group of all atoms lying within a cutoff distance from at least one other atom belonging to the same cluster. We chose a cutoff distance of 3.7 Å (the Ag-Ag correlation distance is 3.55 Å) to define the cluster. Defined in this way, the initial configuration has one large cluster of about 750 atoms and other numerous smaller clusters. At just 2 ps into the damage evolution, the clustering of Ag is lost and the cluster distribution at this point essentially resembles a random configuration. The clusters, however, begin to grow as the network rearranges and recovers its initial connectivity (see Figure 8). The size of the biggest cluster in the network hits its minimum between 1.6 and 2.6 ps (see Figure 5). This is an interesting observation in that it lags behind the time the system has the highest temperature. We observe that the electrical conductivity presumably depends sensitively on the connectivity of the Ag subnetwork, the number, and the structure of the Ag filaments.

We performed *ab initio* calculation of the electronic densities of states (EDOS) of a 648-atom model prepared using the same empirical potential and the damaged snapshots of this model. The size of this model is a compromise between being large enough for damage production and being small enough for an *ab initio* calculation. Our calculations and reference [18] have confirmed that the 648-atom model is statistically similar to the 5184-atom model we discussed above. EDOS calculation is done using plane-wave basis code VASP [27]**-**[29]. Plane waves of up to 350 eV and PAW potentials were used [30]**,**[31].

**Figure 9:**
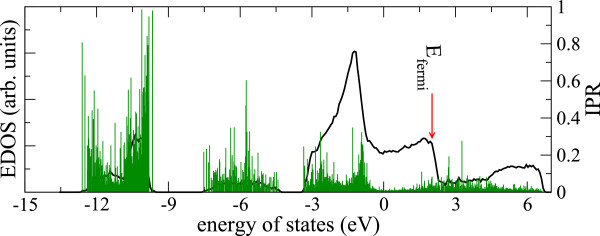
**Density of electronic states and Localization.** The electronic density of states and the IPR, which gauges spatial localization of states of 648-atom models prepared using empirical potential of reference [18]. Fermi level is at 2.01 eV.

**Figure 10:**
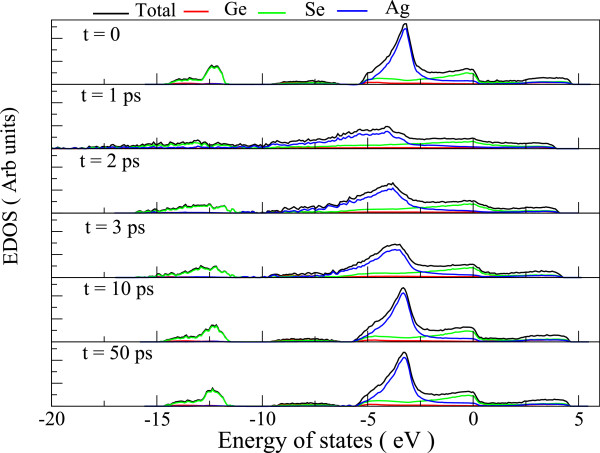
**The evolution and reversibility of electronic structure.** Electronic density of states of six instantaneous configurations of the model at different times with respect to damage event. The model structure and damaged structures were produced using empirical potential, and the electronic structure was calculated for these structures using first principle methods. Note the high degree of reversibility (comparing *t* = 0 and *t* = 50 ps).

EDOS and inverse participation ratio (IPR) (a measure of the spatial localization of the Kohn-Sham electronic states) of our 648-atom model is plotted in Figure 9. A comparison with EDOS from an *ab initio* model [11] reveals that our model lacks an energy gap though there are fewer slightly localized states in the expected gap region. Tracing the structural origin of these states, we note these are p-orbitals localized around Se atoms that are under coordinated with respect to Ge atoms (either one-fold or no Ge coordination). Our calculation on a smaller 100-atom model of [11], prepared using first principles method, also shows that gap states are contributed by Se atoms that are under-coordinated with respect to Ge atoms.We also analyzed the evolution of the electronic structure as the system passes through damage and restoration (see Figure 10). The damaged structures lead to gap states arising from under-coordinated Se atoms. It is interesting to note that the electronic structure recovers very quickly compared to the network itself. The RDF evolution of Figure 7 suggests that although the first peak and first minimum begin to take qualitative shape very early, the network attains similar structural order as the starting configuration as late as 25 ps. The electronic states however show a remarkable healing and reversibility even at 10 ps after the incidence of radiation. This naturally points to an interesting physics underlying the damage recovery that some features of network recover early whereas some others (eg., the Se-Ag correlation) are not recovered even after some equilibration of the system.

Prasai and Drabold [11] have pointed that Se atom bonded with Ag atom contributes to widening the gap. Our work predicts that Se-Ag correlation is lost in the network as a result of radiation-induced damage. The details of the physics of these two separate observations are not fully understood yet.

We have not yet modeled the conductivity of the system. This is complicated by the existence of both ionic and electronic conductivities for some parts of the simulation. Realistic calculations are currently being formulated [32].

## Conclusions

This paper is the first word on the atomistic processes underlying the fascinating experiments and device of Mitkova and coworkers [7]**,**[9]. To fully realize the potential of our approach, many issues such as cell size, composition, details of the modeling of thermal spikes (and subsequent relaxation), and material composition must be explored. Observables like the transport and optical properties should be extracted at representative moments in the simulation. Nevertheless, this work reveals key aspects seen in the experiments including a remarkable reversibility upon annealing. We show that judicious use of the empirical potential of Iyetomi et al. [18] leads to a credible model of the dynamical processes and correctly reproduces many aspects of the material. *Ab initio* methods are an important tool to augment this work and to understand its limitations and the electronic and optical properties.

## Competing interests

The authors declare that they have no competing interests.

## Authors’ contributions

KP and DAD carried out the simulations and jointly wrote the paper. Both authors read and approved the final manuscript.

## References

[B1] Ovshinsky SR Non-Crystalline Materials for Optoelectronics 2004 Bucharest: INOE

[B2] Wuttig M Yamada N Phase-change materials for rewriteable data storage Nat Mater 2007 6 824 832 10.1038/nmat2009 17972937

[B3] Rowlands J Kasap S Amorphous semiconductors usher in digital X-ray imaging Phys Today 1997 50 24 30

[B4] Mitkova M Kozicki MN Silver incorporation in Ge-Se glasses used in programmable metallization cell devices J Non-Crystalline Solids 2002 299–302 1023 1027

[B5] Krecmer P Moulin AM Stephenson RJ Rayment T Welland ME Elliott SR Reversible nanocontraction and dilatation in a solid induced by polarized light Science 1997 277 5333 1799 1802 10.1126/science.277.5333.1799

[B6] Poborchii VV Kolobov AV Tanaka K Photomelting of selenium at low temperature Appl Phys Lett 1999 74 2 215 217 10.1063/1.123297

[B7] Dandamudi P Kozicki M Barnaby H Gonzalez-Velo Y Mitkova M Holbert K Ailavajhala M Yu W Sensors based on radiation-induced diffusion of silver in germanium selenide glasses Nuclear Sci IEEE Trans 2013 60 6 4257 4264

[B8] Mitkova M Butt D Chalcogenide glass ionizing radiation sensor 2013 [http://www.google.com/patents/US8466425] [US Patent 8,466,425]

[B9] Mitkova M Chen P Ailavajhala M Butt D Tenne D Barnaby H Esqueda I Gamma ray induced structural effects in bare and Ag doped Ge–S thin films for sensor application J Non-Crystalline Solids 2013 377 195 199

[B10] Laakkonen J Neiminen RM Computer simulations of radiation damage in amorphous solids Phys Rev B 1990 41 7 3978 3997 10.1103/PhysRevB.41.3978 9994216

[B11] Prasai B Drabold DA Ab initio simulation of solid electrolyte materials in liquid and glassy phases Phys Rev B 2011 83 094202 1 8

[B12] Tafen DN Drabold DA Mitkova M Silver transport in Ge_*x*_Se_*1−x*_*:* Ag materials *ab initio* simulation of a solid electrolyte Phys Rev B 2005 72 054206 1 9

[B13] Gibson JB Goland AN Milgram M Vineyard GH Dynamics of radiation damage Phys Rev 1960 120 4 1229 1253 10.1103/PhysRev.120.1229

[B14] de la Rubia TD Averback RS Benedek R King WE Role of thermal spikes in energetic displacement cascades Phys Rev Lett 1987 59 17 1930 1933 10.1103/PhysRevLett.59.1930 10035371

[B15] Trachenko K Dove MT Artacho E Todorov IT Artacho E Todorov IT Smith W Atomistic simulations of resistance to amorphization by radiation damage Phys Rev B 2006 73 174207 1 15

[B16] Robinson MT Torrens IM Computer simulation of atomic-displacement cascades in solids in the binary-collision approximation Phys Rev B 1974 9 12 5008 5024 10.1103/PhysRevB.9.5008

[B17] Biersack JP Haggmark LG A Monte Carlo computer program for the transport of energetic ions in amorphous targets Nuclear Instrum Mathods 1980 174 257 269 10.1016/0029-554X(80)90440-1

[B18] Iyetomi H Kalia RK PriyaVashishta Incipient phase separation in Ag/Ge/Se glasses: clustering of Ag atoms J Non-Crystalline Solids 2000 262 135 142 10.1016/S0022-3093(99)00692-4

[B19] Drabold DA Topics in the theory of amorphous materials Eur Phys J B 2009 68 1 21 10.1140/epjb/e2009-00080-0

[B20] Plimpton S Fast parallel algorithms for short-range molecular dynamics J Comput Phys 1995 117 1 19 10.1006/jcph.1995.1039

[B21] Dejus R Susman S Volin K Montague G DLPrice Structure of vitreous Ag-Ge-Se J Non-Crystalline Solids 1992 143 162 180

[B22] Kozicki MN Mitkova M Mass transport in chalcogenide electrolyte films- materials and applications J Non-Crystalline Solids 2006 352 567 577 10.1016/j.jnoncrysol.2005.11.065

[B23] Prasai B Chen G Drabold DA Direct *ab initio* molecular dynamic study of ultrafast phase change in Ag-alloyed Ge_*2*_Sb_2_Te_5_ Appl Phys Lett 2013 102 041907 1 4

[B24] Ardondo B Urena MA Piarristeguy A Pradel A Fontana M Homogenous-inhomogenous models of Ag_*x*_(Ge_*0.25*_Se_*0.75*_)_*100−x*_ bulk glasses Phys B 2007 389 77 82 10.1016/j.physb.2006.07.028

[B25] King WE Benedek R Molecular dynamics simulation of low energy displacement cascades in Cu J Nuclear Mater 1983 117 26 35

[B26] Nordlund K Molecular dynamics simulation of ion ranges in the 1–100 keV energy range Comput Mater Sci 1995 3 448 456 10.1016/0927-0256(94)00085-Q

[B27] Kresse G Hafner J Ab initio molecular dynamics for liquid metals Phys Rev B 1993 47 558 561 10.1103/PhysRevB.47.558 10004490

[B28] Kresse G Hafner J Ab initio molecular-dynamics simulation of the liquid-metal-amorphous-semiconductor transition in germanium Phys Rev B 1994 49 14251 14269 10.1103/PhysRevB.49.14251 10010505

[B29] Kresse G Furthmüller J Efficiency of ab-initio total energy calculations for metals and semiconductors using a plane-wave basis set Comput Mater Sci 1996 4 15 50 10.1103/physrevb.54.11169 9984901

[B30] Kresse G Joubert D From ultrasoft pseudopotentials to the projector augmented-wave method Phys Rev B 1999 59 1758 1775

[B31] Blochl PE Projector augmented-wave method Phys Rev B 1994 50 17953 17979 10.1103/PhysRevB.50.17953 9976227

[B32] Abtew TA Zhang M Drabold DA *Ab initio* estimate of the temperature dependence of electrical conductivity in a model disordered material Phys Rev B 2007 19 045212 1 8

